# Supplementary material for: Complexome profiling of the Chlamydomonas psb28 mutant reveals TEF5 as an early PSII assembly factor
Source: Plant Cell. 2025 Mar 17;37(6):koaf055. doi: 10.1093/plcell/koaf055 (PMC12164590; doi:10.1093/plcell/koaf055)
Supplement: koaf055_Supplementary_Data [file koaf055_supplementary_data.zip › psb28_interactive.zip!/plots/intensity/qualityPlots/peptides/_Cre03.g177350.t1.1.html]

### Cre03.g177350.t1.1

Trivial name:   
SeqCoverage [median]: 21.70  
Mass: 41.28 kDa  
  
 

| Sequence | MissedCleavage | Mass [Da] | wtIntensity | muIntensity | Score |
| --- | --- | --- | --- | --- | --- |
| ITAEYTK | 0 | 824.43 | 0.00 | 1707.39 | 73.62 |
| IVALLAAAGR | 0 | 953.60 | 18271.89 | 58492.30 | 160.03 |
| LLADPSFFVK | 0 | 1135.63 | 1878.65 | 0.00 | 69.42 |
| LVWGIDGFR | 0 | 1061.57 | 361.54 | 0.00 | 51.85 |
| SVDSFPADFK | 0 | 1111.52 | 962.74 | 7485.43 | 164.96 |
| VPPGMEPFSLSQR | 0 | 1443.72 | 435.24 | 4218.77 | 66.60 |
| VTPEILQR | 0 | 954.55 | 0.00 | 4307.38 | 109.86 |
| YGLLANK | 0 | 777.44 | 3811.39 | 13725.23 | 122.50 |
| YQIIAGIVEER | 0 | 1289.70 | 2546.08 | 10591.04 | 147.75 |
|  |  |  | 28267.5 | 100527.6 |  |
